# One-Year Comparative Evaluation of Highly Aspherical Lenslets and Horizontally Asymmetric Peripheral Defocus Lenses for Myopia Control in School-Aged Children

**DOI:** 10.3390/life15071119

**Published:** 2025-07-17

**Authors:** Ivana Orešković, Maja Malenica Ravlić, Lana Knežević, Blanka Doko Mandić, Goran Marić, Ante Vukojević, Mia Zorić Geber, Zoran Vatavuk, Ivan Sabol, Jelena Škunca Herman

**Affiliations:** 1 Department of Ophthalmology, Sestre Milosrdnice University Hospital Centre, 10000 Zagreb, Croatia; ivana.oreskovic@kbcsm.hr (I.O.); maja.malenica@kbcsm.hr (M.M.R.); lana.knezevic@kbcsm.hr (L.K.); blanka.doko@kbcsm.hr (B.D.M.); goran.maric@kbcsm.hr (G.M.); ante.vukojevic@kbcsm.hr (A.V.); mia.zoric@kbcsm.hr (M.Z.G.); zoran.vatavuk@kbcsm.hr (Z.V.); 2 Division of Molecular Medicine, Ruđer Bošković Institute (RBI), 10000 Zagreb, Croatia; ivan.sabol@irb.hr

**Keywords:** myopia progression, myopia control, peripheral defocus, axial elongation, highly aspherical lenslets, horizontally asymmetric peripheral defocus lenses, single vision lenses

## Abstract

Purpose: The aim of this study was to compare the one-year efficacy of three spectacle lens designs, highly aspherical lenslets (HALs), horizontally asymmetric peripheral defocus (HAPD) lenses, and standard single vision lenses (SVLs) in slowing myopia progression in school-aged children. Methods: In this prospective, non-randomized study, 57 children, aged 8–17 years, were grouped based on the type of lenses worn: HAL (*n* = 16), HAPD (*n* = 21), or SVL (*n* = 20). Comprehensive ophthalmologic examinations were performed at baseline, 6 months, and 12 months. Outcome measures included spherical equivalent refraction (SER), spherical refraction (SR), cylindrical refraction (CR), and axial length (AL). Data were analyzed using non-parametric tests with significance set at *p* < 0.05. Results: All groups showed some progression in SER and AL over 12 months. The HAL group demonstrated the smallest median SER change (−0.3 D), compared to HAPD (−0.5 D) and SVL (−0.4 D), though group differences were not statistically significant (*p* = 0.111). Axial elongation was significantly lower in the HAL group (0.1 mm, IQR: 0.0–0.2 mm) compared to HAPD and SVL (both 0.2 mm, *p* < 0.0001). CR remained stable in all groups, with no clinically meaningful changes. The HAPD groups showed no advantages over SVL in any parameter. Conclusions: Among the three lens types studied, HAL lenses were the most effective in reducing both refractive and axial myopia progression over 12 months. These findings support their use as a reliable intervention in pediatric myopia control.

## 1. Introduction

Myopia prevalence increases progressively throughout childhood, with the highest incidence occurring during and after puberty, coinciding with the adolescent growth phase of the eye [1]. Recent global data indicate that the proportion of children and adolescents affected by myopia rose from 24.32% in 1990 to 35.81% in 2023, with projections estimating it may approach 40% by 2050, particularly among adolescents, females, and individuals living in urban environments [2]. Axial elongation during childhood is a major contributor to the development of more severe myopia later in adolescence and adulthood. Elevated levels of myopia are associated with a substantially increased risk of long-term, sight-threatening complications such as myopic macular degeneration and retinal detachment [3,4]. As a result, early identification and effective myopia control strategies in the pediatric population are essential to reducing the global burden of visual impairment. A key optical mechanism implicated in myopia progression is peripheral hyperopic defocus, where peripheral light rays focus behind the retinal plane. This state is believed to trigger compensatory axial elongation. Myopia control interventions aim to counter this by inducing peripheral myopic defocus—shifting the peripheral focal point in front of the retina—to slow ocular growth [5]. Spectacle lenses for myopia control apply this principle through various optical designs. Highly aspherical lenslets (HALs) incorporate hundreds of small lenslets arranged in a concentric and uniform pattern across the lens surface, generating consistent myopic defocus in all directions while maintaining central visual clarity [6]. Horizontal asymmetrical peripheral defocus (HAPD) lenses, a type of horizontal perifocal lenses, use a horizontally asymmetric optical design that provides clear central vision while inducing targeted myopic defocus along the horizontal meridian of the peripheral retina [7]. Previous clinical trials have shown that both HALs and horizontal perifocal lenses are effective in reducing the rate of myopia progression in children [6,8]. However, the effectiveness of HAPD lenses, as a subtype of horizontal perifocal designs, remains less well-documented. In particular, there is a lack of published data on their optical mechanism of action and comparative clinical efficacy. Since HAPD lenses are available on our market, we decided to investigate their impact. Further studies are needed to evaluate their role in real-world pediatric populations, which is essential for evidence-based decision-making in myopia management. Given the increasing global prevalence of myopia and the importance of early intervention, evaluating the relative effectiveness of different spectacle lens designs is of high clinical relevance. Therefore, this study aimed to assess and compare the efficacy of highly aspherical lenslets and horizontal asymmetrical peripheral defocus lenses in slowing myopia progression in children, in comparison to standard single vision lenses as a control.

## 2. Materials and Methods

This comparative, non-randomized, prospective study was conducted at the Department of Ophthalmology, University Hospital Centre “Sestre milosrdnice” in Zagreb, Croatia, between 2024 and 2025. A total of 57 Caucasian children, aged between 8 and 17 years, were enrolled. All participants were diagnosed with myopia according to the definition of the International Myopia Institute (IMI) [9]. For statistical analysis, only the right eye was included, and then interpreted in the Section 3. Data for the left eye were analyzed separately and are provided in the Supplementary Materials.

### 2.1. Ethical Considerations

The study was approved by the Ethics Committee of the University Hospital Centre “Sestre milosrdnice,” which operates in accordance with the principles of the International Conference on Harmonisation—Good Clinical Practice (ICH-GCP) and the Declaration of Helsinki. The approval was granted under Class 003-06/25-03/014; Reg. No.: 251-29-11/3-25-10. Written informed consent was obtained from the parents or legal guardians of all participants prior to inclusion in the study.

### 2.2. Participants

Eligible participants were Caucasian children aged 8 to 17 years with a spherical equivalent refractive (SER) error ranging from −1.00 to −6.00 diopters (D) and a maximum cylindrical refractive error of −1.75 D. They were regular users of one of three specific types of spectacle lenses. All participants were required to wear their prescribed lenses for a minimum of 12 h per day throughout the study period, as confirmed by parent-reported adherence. Individuals were excluded if they presented with ocular pathology other than myopia, had a history of ocular surgery, systemic diseases affecting the eye, had spherical refractive errors (SR) greater than −1.00 D or less than −6.00 D, or cylindrical refractive (CR) errors greater than 1.75 D.

Participants were categorized into three groups based on the type of spectacle lenses they were wearing at the time of enrollment. The first group included users of highly aspherical lenslets (HALs) designed for myopia control. The second group consisted of children wearing spectacle lenses with horizontal asymmetrical peripheral defocus (HAPD), a type of horizontal perifocal lenses. The third group comprised users of standard single-vision spectacle lenses (SVLs).

Furthermore, all participants were subdivided according to axial length (AL) (<25 mm and ≥25 mm), SER (−1.00 D to −4.00 D and −4.00 D to −6.00 D), and age (8–12 years and 13–17 years) to enable subgroup analysis and evaluate potential differences in treatment response across these parameters. In addition, for categorical analysis, participants were classified based on the degree of AL and SER change over 12 months. AL change was categorized as decrease (<0 mm), stable (0–0.20 mm), or increase (>0.20 mm). SER change was classified as myopia progression (worse than −0.50 D), stable (−0.50–0 D), or myopia improvement (>0 D). Clinical data for all participants were extracted from the hospital information system and analyzed in an anonymized form.

### 2.3. Clinical Examinations

All participants underwent comprehensive ophthalmologic examinations at baseline and six-month intervals throughout a minimum follow-up period of one year. Each examination was non-invasive and included a detailed medical and/or parent-reported history, assessment of distance and near visual acuity using standardized LogMAR charts under miosis, slit-lamp biomicroscope of the anterior segment under both miosis and pharmacologically induced mydriasis, fundus examination, cycloplegic retinoscopy, and AL measurement performed using the IOL Master 700 (Carl Zeiss Meditec, Jena, Germany). All assessments were conducted by the same experienced examiner using standardized procedures to ensure consistency.

### 2.4. Cycloplegia Protocol

Cycloplegia was induced in accordance with IMI guidelines. To minimize discomfort, one drop of Tetracaine 0.5% (Tetrakain^®^, Théa, Clermont-Ferrand, France) was instilled into each eye. One minute later, a drop of Tropicamide 1% (Mydriacyl^®^, Alcon, Geneva, Switzerland) was administered, followed by a second drop five minutes later. Thirty minutes after the first instillation of Tropicamide, cycloplegic retinoscopy and axial length measurements were performed.

### 2.5. Spectacle Lens Characteristics

The HALs used in this study were Essilor Stellest, manufactured by Essilor, part of the Essilor Luxottica group [10]. The lenses are composed of Airwear 1.59 polycarbonate, a material known for its high impact resistance, lightweight properties, and inherent ultraviolet (UV) protection [11]. This material offers enhanced safety and comfort, making it especially suitable for pediatric use. All lenses were coated with Crizal Kids, an antireflective and multipurpose coating that protects against glare, scratches, smudges, and dust [11,12]. Optically, the Stellest lens design features a central optical zone approximately 9 mm in diameter, which provides clear central vision and corrects distance refractive error. Surrounding this central zone are 11 concentric rings containing a total of 1021 highly aspherical, contiguous lenslets, each with a diameter of approximately 1.12 mm. These lenslets do not function as simple additional power zones, but instead create a three-dimensional volume of myopic defocus (VoMD), a focused light volume in front of the retina, acting as a signal to slow down axial eye elongation. The inter-ring spaces preserve single-vision correction and contribute to maintaining clarity in peripheral perception. The overall optical design has been optimized to minimize negative impact on visual acuity, contrast sensitivity, and binocular vision [10].

The HAPD lenses used in this study were Rodenstock MyCon™, manufactured by Rodenstock GmbH (Munich, Germany) [13]. These lenses are made from impact-resistant organic plastic and are available in multiple refractive indices (1.50, 1.60, 1.67, and 1.74), which allows for thinner lens designs across a broad range of prescriptions [14]. MyCon™ lenses utilize Horizontal Asymmetrical Peripheral Defocus (HAPD™) technology, in which an uneven defocus is created along the horizontal meridian in the peripheral zones. This configuration provides clear central distance correction while inducing peripheral myopic defocus aimed at slowing axial eye growth. The central zone of the lens corrects distance refractive error without compromising image quality in the central visual field. Approximately +2.00 D of defocus is induced on the nasal side of the lens, while the temporal side provides around +2.50 D. This asymmetry corresponds to known anatomical differences in peripheral refraction and may contribute to increased effectiveness in myopia control [15].

### 2.6. Data Management

All collected data were anonymized and stored in a secure, password-protected institutional database. No personally identifying information was included in the final analytical dataset.

### 2.7. Outcome Measures

The primary outcome measures assessed at each visit included best corrected visual acuity (BCVA), objective cycloplegic refractive error including SER, SR, and CR as well as AL of the eye.

### 2.8. Statistical Analysis

Statistical analysis was performed using MedCalc v23.2 (MedCalc Software, Ostend, Belgium). The Kolmogorov–Smirnov test was used to assess the normality of the distribution. Categorical data were analyzed using a Chi-square test. The Friedman test (non-parametric repeated measures ANOVA) was used to investigate the changes in each parameter through time for each glass type. Differences between the 12-month timepoint and baseline between different glasses groups were compared with the Kruskal–Wallis test. *p* values of less than 0.05 were considered significant. Categorical analyses of AL and SER changes were also conducted based on predefined clinical thresholds.

## 3. Results

A total of 57 school-aged children, aged 8 to 17 years, were enrolled in the study, with a median age of 12 years (interquartile range (IQR): 10.0–14.0). The overall gender distribution was balanced (49.1% female). The HAL group included a greater proportion of younger participants (62.5% aged 8–12 years) and males (75%) compared to the HAPD (38.1% males) and SVL (45% males) groups (Table 1).

**Figure 1:**
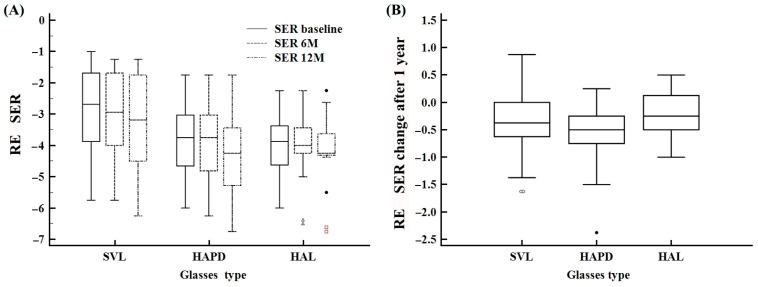
Changes in spherical equivalent refraction (SER) of the right eye (RE) over time and between treatment groups. (**A**) Distribution of SER values at baseline, 6 months, and 12 months across the SVL, HAPD, and HAL groups. (**B**) Total SER change after 12 months in each group.

At baseline, right eye (RE) SER significantly differed among groups (Kruskal–Wallis *p* = 0.0138), with differences persisting at 6 months (*p* = 0.0273) and marginally insignificant at 12 months (*p* = 0.0599) (Figure 1). Similar findings were observed for the left eye (LE) (Supplementary Table S1 and Figure S1). Over time, RE SER changed significantly within all groups (Friedman test *p* < 0.0001 for HAPD and HAL; *p* = 0.0325 for SVL), except for SVL LEs (*p* = 0.0911). As shown in Figure 1A, the HAL group showed relatively stable RE SER values over time, compared to more variable trends in SVL and HAPD groups. Although group differences at 12 months were not statistically significant (Kruskal–Wallis *p* = 0.115 for right and *p* = 0.136 for left eyes), the HAL group displayed the narrowest IQR, indicating a more clustered distribution of RE SER values (Figure 1B; Table 2).

**Figure 2:**
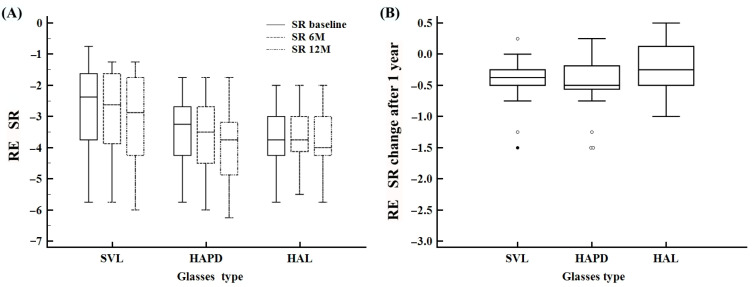
Changes in spherical refraction (SR) of the right eye (RE) over time and between treatment groups. (**A**) Distribution of SR values at baseline, 6 months, and 12 months across the SVL, HAPD, and HAL groups. (**B**) Total SR change after 12 months by treatment group.

RE SR followed a similar trend to SER (Table 2). Kruskal–Wallis testing showed statistically significant differences among groups at baseline (*p* = 0.0174) and 6 months (*p* = 0.0443), but not at 12 months (*p* = 0.0799) (Figure 2A). Significant within-group changes over time were found in all groups (Friedman test *p* < 0.0001 for SVL and HAPD, *p* = 0.0325 for HAL). The HAL group showed the most consistent progression pattern with less variability, while HAPD demonstrated greater interindividual differences (Figure 2A). Although the 12-month median SR progression in HAL and SVL was similar, no statistically significant difference was found between them. However, the HAL group demonstrated a narrower IQR, suggesting less interindividual variability. This observation did not reach statistical significance (Kruskal–Wallis *p* = 0.115) and should be interpreted with caution (Figure 2B; Table 2). A similar pattern was seen in left eye measurements as well (Supplementary Table S1).

**Figure 3:**
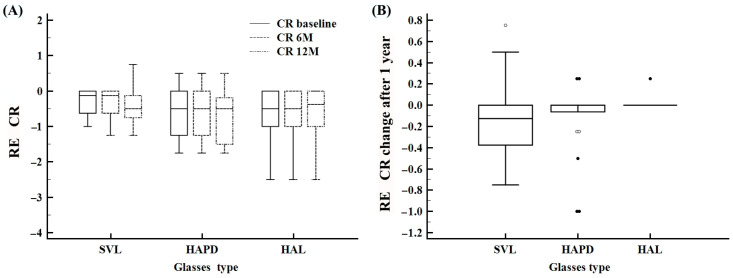
Changes in cylindrical refraction (CR) of the right eye (RE) over time and between treatment groups. (**A**) Distribution of CR values at baseline, 6 months, and 12 months across the SVL, HAPD, and HAL groups. (**B**) Total CR change after 12 months by treatment group.

RE CR remained stable across all groups over 12 months, with a slight downward trend observed only in the SVL group (Figure 3A). Kruskal–Wallis analysis revealed a small but statistically significant group difference (*p* = 0.048), with post hoc Dunn testing showing significance only between HAL and SVL. However, these differences did not translate into clinically meaningful changes in cylindrical refraction in any group (Figure 3B).

**Figure 4:**
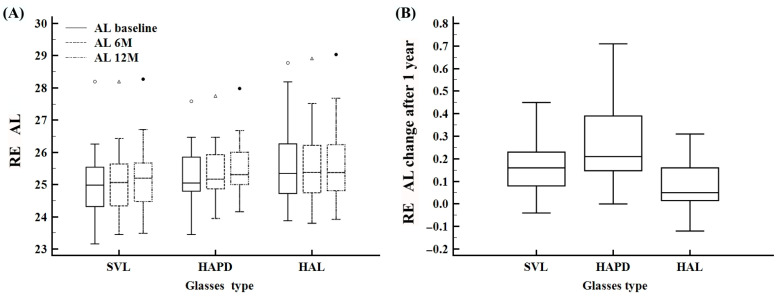
Changes in axial length (AL) of the right eye (RE) over time and between treatment groups. (**A**) Distribution of AL values at baseline, 6 months, and 12 months across the SVL, HAPD, and HAL groups. (**B**) Total AL change after 12 months by treatment group.

AL increased significantly over 12 months in all groups (Friedman test *p* < 0.0001 for RE in HAPD and HAL; *p* = 0.0059 for RE in SVL) (Figure 4A). At 12 months, the HAL group showed the lowest RE median axial elongation (0.1 mm, IQR: 0.0–0.2), while both HAPD and SVL showed a median increase of 0.2 mm. At 12 months, group comparisons revealed statistically significant differences in RE and LE AL elongation (Kruskal–Wallis *p* = 0.0067 for RE; *p* = 0.0031 for LE), primarily driven by differences between the HAL group and the other two treatment groups (HAPD and SVL), as confirmed by post hoc testing (Figure 4B). At baseline, RE AL values were slightly higher in the HAL group compared to others (Table 2), and a greater proportion of eyes with RE AL ≥ 25 mm was observed in HAL and HAPD (Table 1). LE AL changes followed a similar trend (Supplementary Table S1 and Figure S2). No statistically significant differences in 12-month AL changes were found between subgroups defined by age, sex, baseline SER, or initial AL category (RE or LE), based on Mann–Whitney testing.

To further explore treatment effects, participants were categorized by AL and SER changes at 12 months. AL change was classified as increase (>0.20 mm), stable (0–0.20 mm), or decrease (<0 mm), while SER change was defined as myopia progression (worse than −0.50 D), stable (−0.50–0 D), or myopia improvement (>0 D). For REs, the distribution of AL change significantly differed between treatment groups (Chi-square = 9.888, *p* = 0.0424), while for LEs this difference approached statistical significance (*p* = 0.0537). No statistically significant differences in the distribution of SER change were observed between groups for either right (*p* = 0.5272) or left eyes (*p* = 0.4195). Detailed categorical distributions are presented in Table 3 and Supplementary Table S2.

## 4. Discussion

This study aimed to evaluate the one-year efficacy of three different types of spectacle lenses—HAL, HAPD, and SVL—in controlling myopia progression in school-aged children, focusing on right eye measurements of SER and AL as key outcomes. Although all groups demonstrated myopia progression over 12 months, the HAL group showed the most favorable outcome in terms of axial elongation. In terms of SER, changes in the HAL group were numerically smaller and more stable throughout follow-up, and the group exhibited the narrowest IQR in SER progression, indicating lower interindividual variability. While the difference in 12-month SER progression among groups did not reach statistical significance (*p* = 0.115), the HAL group showed the smallest median SER change (−0.3 D), followed by the SVL (−0.4 D) and HAPD (−0.5 D) groups. These results are broadly consistent with findings from previous trials. For example, Bao et al. reported a mean 12-month SER progression of −0.27 D in children wearing HAL lenses versus −0.81 D in those wearing SVL. The narrower IQR observed in our HAL group aligns with the greater interindividual consistency described in earlier HAL studies [6,8]. Comparable results have also been observed with other optical designs. In a recent European retrospective cohort study by Lembo et al., both DIMS (Defocus Incorporated Multiple Segments) and HAL lenses demonstrated similar efficacy in slowing myopia progression over two years. At one year, the mean SER change was −0.34 D in the DIMS group and −0.30 D in the HAL group. After two years, HAL and DIMS lenses showed similar axial elongation (0.32 mm vs. 0.29 mm). The study concluded that both lens types provided equivalent outcomes in European pediatric populations, supporting their interchangeable use clinical use in clinical practice [16]. In contrast, the HAPD group in our study exhibited the largest myopia progression, though this difference did not reach clinical or statistical significance. This finding differs from some earlier studies that have suggested a benefit of peripheral defocus lenses with horizontal treatment zones. These two types of lenses share the same design principle of horizontal asymmetrical peripheral defocus (HAPD). For instance, Tarutta et al. reported cumulative mean SER progressions of −0.38 D after 12–18 months, −0.78 D after 2 years, −0.99 D after 3 years, and −1.16 D after 4–5 years with perifocal lenses. The discrepancy may be partly explained by differences in the number of participants, age group, and duration of the study [8]. It is possible that the long-term effect of the lenses in the study by Tarutta et al. may be more favorable. Another limited and uneven peripheral effect of such asymmetric designs comes from a Portuguese study by Silva-Leite et al., who assessed the peripheral optical profile of perifocal lenses in children with progressing myopia. Their results showed significant myopic defocus only in the nasal retina (approximately −0.42 D at 25° eccentricity), while changes in the temporal retina were not statistically significant. Additionally, the measured astigmatic myopic peripheral defocus was lower than manufacturer-reported values, suggesting a weaker than expected treatment signal [17]. Although the observed SER progression in the SVL group in our study (−0.4 D) appears lower than values typically reported in East Asian populations (−0.82 D/year) or in European populations (−0.55 D/year) [18], this may reflect demographic or environmental differences (e.g., outdoor time, working near a computer), as well as possible variations in lens wear compliance.

Spherical refraction (SR) followed a similar trend to SER, with the HAL group showing limited median progression (−0.3 D) and the narrowest IQR, indicating less variability in response. While these findings do not confirm a statistically significant treatment effect, the consistency observed in the HAL group may warrant further investigation.

Axial length, a key anatomical marker of myopia progression, revealed statistically and clinically relevant intergroup differences. Axial elongation was significantly lower in the HAL group (median 0.1 mm, IQR: 0.0–0.2) compared to both HAPD and SVL groups (each with median 0.2 mm). These results are consistent with the proposed mechanism of HAL lenses, which are designed to generate stronger and more consistent peripheral myopic defocus to inhibit axial elongation. Bao et al. reported a mean one-year AL progression of 0.13 mm in children wearing HAL lenses versus 0.36 mm in those wearing SVL, while in the Lembo et al. study, AL increased by 0.15 mm in HAL wearers [6,16]. Longitudinal studies confirm that HAL lenses reduce AL growth by approximately 0.3–0.7 mm over multi-year (2–5 years) periods [19]. In a 2-year randomized trial, the mean (SE) increase in AL was reported as +0.34 (0.03) mm in the HAL group and +0.69 (0.04) mm in the SVL group. Annualized, this corresponds to approximately +0.17 mm/year for HAL and +0.35 mm/year for SVLs. In a 2-year randomized clinical trial, Bao et al. reported that the HAL group showed a significantly smaller increase in AL compared to the SVL group, with a mean difference of 0.35 mm (standard error: 0.05 mm; 95% CI: 0.23–0.47; *p* < 0.001). In a higher-risk subgroup of younger children (aged 8–12 years), the mean difference was 0.18 mm (SE: 0.05 mm; 95% CI: 0.06–0.30; *p* = 0.001), indicating a consistent treatment effect in favor of HAL lenses [20]. Despite including a higher proportion of younger children in the HAL group (62.5% aged 8–12), who are typically at greater risk of rapid myopic progression, axial growth remained minimal. This supports the potential value of HAL lenses even in higher-risk subgroups. In contrast, HAPD lenses did not show a measurable benefit over SVL in terms of AL elongation, with both groups showing a median increase of 0.2 mm. This further suggests that the asymmetric horizontal defocus design in HAPD lenses may be suboptimal for achieving meaningful axial control in clinical settings. In Tarutta’s study, the average axial elongation was reported as +0.05 mm after 6 months, +0.11 mm after 12–18 months, +0.22 mm after 2 years, +0.36 mm after 3 years, and +0.46 mm after 4–5 years. These data indicate that asymmetric perifocal lenses in that study were more effective in slowing axial eye growth compared to the HAPD lenses in our study [8]. Cylindrical refraction remained largely unchanged across all groups. Although a small but statistically significant difference in CR was detected between HAL and SVL, this finding lacked clinical relevance and likely reflects measurement variability rather than a true optical effect. Overall, the results suggest that none of the tested lenses had a meaningful impact on astigmatic components.

Finally, while differences in age and baseline refractive error across groups were minimal, the observed patterns in younger children, especially those wearing HAL lenses, further reinforce the potential protective effect of this lens design.

## 5. Conclusions

HAL lenses demonstrated numerically the most favorable outcomes in terms of slowing both refractive progression and axial elongation over a one-year period. While differences in SER progression did not reach statistical significance, the HAL group showed a more consistent treatment response and the lowest axial elongation among the three lens types. These findings are in agreement with previous studies and suggest that HAL lenses may offer a clinically meaningful benefit in myopia control, particularly in younger children who are at greater risk for faster progression. The limited efficacy observed with HAPD lenses in our cohort highlights the potential influence of lens design asymmetry and underscores the need for further research to optimize peripheral defocus strategies. Overall, the results support the potential utility of HAL lenses in managing pediatric myopia while emphasizing the importance of individualized treatment selection based on patient age, baseline refractive status, and expected risk of progression.

## 6. Study Limitations

This study has several limitations that should be considered when interpreting the results. First, the sample size was relatively small, particularly when stratified by lens type and age subgroups, which may have limited the statistical power to detect subtle differences between groups. Second, the non-randomized study design introduces potential selection bias, as lens allocation was based on patient and parent preference rather than random assignment. This may have resulted in baseline imbalances, such as the higher proportion of younger children and males in the HAL group, which could have influenced outcomes. In particular, younger age is associated with faster myopia progression, potentially biasing the HAL group toward worse outcomes. However, despite this higher-risk profile, the HAL group still demonstrated the most favorable treatment effect, suggesting robustness of the lens design even in more susceptible subgroups. Third, although all children were followed for a full 12 months, variations in compliance, wearing time, and environmental factors (time spent outdoors, near work) were not controlled or systematically recorded, which could have affected the progression rates. Fourth, the study relied on central optical measurements and did not include peripheral refraction mapping or higher-order aberration analysis, which could have provided further insight into the optical mechanisms of action of different lens designs. Despite these limitations, the study provides valuable real-world comparative data on the effectiveness of three clinically relevant spectacle lens designs in managing myopia progression in school-aged children. Additionally, although the HAL group showed numerically favorable outcomes, its smaller size and younger age profile may limit the generalizability of these findings.

## Figures and Tables

**Table 1:** Basic characteristics of respondents by groups.

	Total (*n* = 57)	SVL (*n* = 20)	HAPD (*n* = 21)	HAL (*n* = 16)
Gender				
F	28 (49.1%)	11 (55%)	13 (61.9%)	4 (25%)
M	29 (50.9%)	9 (45%)	8 (38.1%)	12 (75%)
Age, median (IQR)	12 (10–14)	12.5 (11–14.5)	12 (10.8–13.3)	11 (9.5–14)
Age group				
8−12	31 (54.4%)	10 (50%)	11 (52.4%)	10 (62.5%)
13–17	26 (45.6%)	10 (50%)	10 (47.6%)	6 (37.5%)
Right eye baseline SER, median (IQR)	−3.5 (−4.5–−2.6)	−2.7 (−3.9–−1.7)	−3.8 (−4.7–−3.0)	−3.9 (−4.6–−3.4)
Miopia severity group, right eye				
−1 to −4	39 (68.4%)	17 (85%)	11 (52.4%)	11 (68.8%)
−4.1 to −6	18 (31.6%)	3 (15%)	10 (47.6%)	5 (31.3%)
Right eye baseline AL, median (IQR)	25.1 (24.7–25.8)	25.0 (24.3–25.5)	25.1 (24.8–25.9)	25.4 (24.7–26.3)
Axial length group, right eye				
<25 mm	28 (49.1%)	11 (55%)	10 (47.6%)	7 (43.8%)
>25 mm	29 (50.9%)	9 (45%)	11 (52.4%)	9 (56.3%)
Left eye baseline SER, median (IQR)	−3.3 (−4.1–−2.3)	−2.4 (−3.2–−2)	−3.5 (−4.6–−3.1)	−3.8 (−4.5–−2.9)
Left eye baseline AL, median (IQR)	25.1 (24.7–25.6)	24.9 (24.5–25.3)	25.2 (24.8–25.7)	25.2 (24.7–26.4)

**Table 2:** Summary of refractive (SER, SR, CR) and biometric (AL) parameters of the right eye (RE) of each participant at baseline, 6 months, and 12 months for the total sample and by treatment group.

RE	Total (*n* = 57)			SVL (*n* = 20)			HAPD (*n* = 21)			HAL (*n* = 16)		
	Min−Max	Mean ± SD	Median (IQR)	Min−Max	Mean ± SD	Median (IQR)	Min−Max	Mean ± SD	Median (IQR)	Min−Max	Mean ± SD	Median (IQR)
SER baseline	−6.0–−1.0	−3.5 ± 1.3	−3.5 (−4.5–−2.5)	−5.7–−1.0	−2.8 ± 1.3	−2.6 (−3.8–−1.6)	−6.0–−1.7	−3.7 ± 1.2	−3.7 (−4.6–−3.0)	−6.0–−2.2	−4.0 ± 1.1	−3.8 (−4.6–−3.3)
SER 6 M	−6.5–−1.2	−3.6 ± 1.3	−3.7 (−4.3–−2.6)	−5.7–−1.2	−2.9 ± 1.3	−2.9 (−4.0–−1.6)	−6.2–−1.7	−3.9 ± 1.3	−3.7 (−4.8–−3.0)	−6.5–−2.2	−4.0 ± 1.1	−4.0 (−4.2–−3.4)
SER 12 M	−6.7–−1.2	−3.9 ± 1.4	−4.0 (−5.0–−2.9)	−6.2–−1.2	−3.2 ± 1.5	−3.1 (−4.5–−1.7)	−6.7–−1.7	−4.2 ± 1.4	−4.2 (−5.2–−3.4)	−6.7–−2.2	−4.2 ± 1.2	−4.2 (−4.3–−3.6)
SR baseline	−5.7–−0.7	−3.2 ± 1.2	−3.2 (−4.0–−2.1)	−5.7–−0.7	−2.6 ± 1.3	−2.3 (−3.7–−1.6)	−5.7–−1.7	−3.4 ± 1.0	−3.2 (−4.2–−2.6)	−5.7–−2.0	−3.6 ± 1.0	−3.7 (−4.2–−3.0)
SR 6 M sfera	−6.0–−1.2	−3.3 ± 1.2	−3.2 (−4.2–−2.5)	−5.7–−1.2	−2.7 ± 1.3	−2.6 (−3.8–−1.6)	−6.0–−1.7	−3.6 ± 1.2	−3.5 (−4.5–−2.6)	−5.5–−2.0	−3.7 ± 1.0	−3.7 (−4.1–−3.0)
SR 12 M sfera	−6.2–−1.2	−3.6 ± 1.3	−3.5 (−4.5–−2.6)	−6.0–−1.2	−3.0 ± 1.4	−2.8 (−4.2–−1.7)	−6.2–−1.7	−3.9 ± 1.2	−3.7 (−4.8–−3.1)	−5.7–−2.0	−3.8 ± 1.1	−4.0 (−4.2–−3.0)
CR baseline	−2.0–0.50	−0.5 ± 0.5	−0.5 (−0.8–0.0)	−1.0–0.0	−0.3 ± 0.3	−0.1 (−0.6–0.0)	−1.7–0.50	−0.6 ± 0.6	−0.5 (−1.2–0.0)	−2.0–0.0	−0.5 ± 0.6	−0.5 (−0.7–0.0)
CR 6 M cilindar	−2.0–0.50	−0.5 ± 0.6	−0.5 (−1.0–0.0)	−1.2–0.0	−0.3 ± 0.4	−0.1 (−0.6–0.0)	−1.7–0.50	−0.6 ± 0.6	−0.5 (−1.2–0.0)	−2.0–0.0	−0.5 ± 0.6	−0.5 (−0.6–0.0)
CR 12 M cilindar	−2.0–0.75	−0.5 ± 0.6	−0.5 (−1.0–0.0)	−1.2–0.75	−0.4 ± 0.4	−0.5 (−0.7–−0.1)	−1.7–0.50	−0.7 ± 0.6	−0.5 (−1.5–−0.1)	−2.0–0.0	−0.5 ± 0.6	−0.2 (−0.7–0.0)
AL baseline	23.1–28.7	25.2 ± 1.1	25.0 (24.6–25.8)	23.1–28.1	25.0 ± 1.0	24.9 (24.3–25.5)	23.4–27.5	25.2 ± 0.9	25.0 (24.7–25.8)	23.8–28.7	25.6 ± 1.3	25.3 (24.7–26.2)
AL 6 M	23.4–28.9	25.3 ± 1.0	25.1 (24.7–25.8)	23.4–28.1	25.1 ± 1.0	25.0 (24.3–25.6)	23.9–27.7	25.4 ± 0.8	25.1 (24.8–25.9)	23.8–28.9	25.5 ± 1.3	25.3 (24.7–26.2)
AL 12 M	23.4–29.0	25.4 ± 1.0	25.2 (24.8–25.9)	23.4–28.2	25.2 ± 1.0	25.2 (24.4–25.6)	24.1–27.9	25.5 ± 0.8	25.3 (25.0–26.0)	23.9–29.0	25.6 ± 1.3	25.3 (24.8–26.2)
SER difference 12 M	−1.6–0.50	−0.4 ± 0.4	−0.3 (−0.7–0.0)	−1.6–0.25	−0.4 ± 0.4	−0.4 (−0.7–−0.1)	−1.5–0.12	−0.5 ± 0.4	−0.5 (−0.7–−0.1)	−1.0–0.50	−0.1 ± 0.4	−0.3 (−0.5–0.12)
SR difference 12 M	−1.5–0.50	−0.3 ± 0.4	−0.2 (−0.5–0.0)	−1.5–0.25	−0.4 ± 0.4	−0.3 (−0.5–−0.2)	−1.5–0.25	−0.4 ± 0.4	−0.5 (−0.5–−0.1)	−1.0–0.50	−0.2 ± 0.4	−0.3 (−0.5–0.12)
CR difference 12 M	−1.0–0.75	−0.0 ± 0.2	0.0 (−0.2–0.0)	−0.7–0.75	−0.1 ± 0.3	−0.1 (−0.3–0.0)	−1.0–0.25	−0.1 ± 0.3	0.0 (−0.0–0.0)	0.0–0.25	0.01 ± 0.0	0.0 (0.0–0.0)
AL difference 12 M	−0.1–0.71	0.17 ± 0.1	0.16 (0.05–0.26)	−0.0–0.45	0.17 ± 0.1	0.16 (0.08–0.23)	0.0–0.71	0.25 ± 0.1	0.21 (0.14–0.39)	−0.1–0.31	0.07 ± 0.1	0.1 (0.0–0.2)

**Table 3:** Categorical distribution of axial length (AL) and spherical equivalent refraction (SER) changes in right eyes (RE) at 12 months by treatment group.

RE	SVL (*n* = 20)	HAPD (*n* = 21)	HAL (*n* = 16)	Total	Chi-Square
AL decrease	2 (10%)	0 (0%)	4 (25%)	6 (10.5%)	*p* = 0.0424
AL increase	7 (35%)	11 (52.4%)	2 (12.5%)	20 (35.1%)	
Al stable	11 (55%)	10 (47.6%)	10 (62.5%)	31 (54.4%)	
Myopia improve	5 (25%)	5 (23.8%)	7 (43.8%)	17 (29.8%)	*p* = 0.5272
Myopia increase	7 (35%)	9 (42.9%)	3 (18.8%)	19 (33.3%)	
Myopia stable	8 (40%)	7 (33.3%)	6 (37.5%)	21 (36.8%)	

## Data Availability

The raw data are available upon reasonable request from the corresponding author.

## Supplementary Materials

The following supporting information can be downloaded at: https://www.mdpi.com/article/10.3390/life15071119/s1, Figure S1:Changes in spherical equivalent refraction (SER) of the left eye (LE) over time and between treatment groups. (A) Distribution of SER values at baseline, 6 months, and 12 months across the SVL, HAPD, and HAL groups. (B) Total SER change after 12 months in each group. Figure S2: Changes in axial length (AL) of the left eye (LE) over time and between treatment groups. (A) Distribution of AL values at baseline, 6 months, and 12 months across the SVL, HAPD, and HAL groups. (B) Total AL change after 12 months by treatment group. Table S1: Summary of refractive (SER, SR, CR) and biometric (AL) parameters of the left eye (LE) of each participant at baseline, 6 months, and 12 months for the total sample and by treatment group. Table S2: Categorical distribution of axial length (AL) and spherical equivalent refraction (SER) changes in left eyes (LE) at 12 months by treatment group.
