# Supplementary material for: One-Year Comparative Evaluation of Highly Aspherical Lenslets and Horizontally Asymmetric Peripheral Defocus Lenses for Myopia Control in School-Aged Children
Source: Life (Basel). 2025 Jul 17;15(7):1119. doi: 10.3390/life15071119 (PMC12300819; doi:10.3390/life15071119)
Supplement: Supplementary file 1 [file life-15-01119-s001.zip › life-3716327-supplementary.pdf]

LE	Total (n=57)			SVL (n=20)			HAPD (n=21)			HAL (n=16)		
	Min-Max	Mean±SD	Median (IQR)	Min-Max	Mean±SD	Median (IQR)	Min-Max	Mean±SD	Median (IQR)	Min-Max	Mean±SD	Median (IQR)
SER baseline	-5.7 - -1.0	-3.0±1.1	-3.0 (-4.0 - -2.0)	-5.7 - -1.0	-2.4±1.1	-2.0 (-2.8 - -1.8)	-5.2 - -1.0	-3.2±1.0	-3.0 (-4.1 - -2.5)	-5.5 - -1.5	-3.4±1.0	-3.3 (-4.0 - -2.8)
SER 6M	-5.7 - -1.0	-3.1±1.2	-3.0 (-3.8 - -2.2)	-5.7 - -1.0	-2.5±1.1	-2.2 (-3.0 - -1.8)	-5.7 - -1.0	-3.4±1.1	-3.2 (-4.5 - -2.8)	-5.5 - -1.5	-3.5±1.1	-3.7 (-4.1 - -2.7)
SER 12M	-6.0 - -1.0	-3.3±1.2	-3.2 (-4.2 - -2.4)	-6.0 - -1.0	-2.7±1.2	-2.5 (-3.3 - -1.6)	-6.0 - -1.5	-3.7±1.2	-3.5 (-4.8 - -2.9)	-5.5 - -1.5	-3.5±1.1	-3.6 (-4.3 - -2.8)
SR baseline	-1.7 - 0.50	-0.4±0.5	-0.1 (-0.7 - 0.0)	-1.0 - 0.0	-0.2±0.3	0.0 (-0.5 - 0.0)	-1.5 - 0.50	-0.5±0.6	-0.3 (-1.0 - 0.0)	-1.7 - 0.0	-0.4±0.5	0.0 (-0.6 - 0.0)
SR 6M	-1.7 - 0.50	-0.4±0.5	-0.2 (-1.0 - 0.0)	-1.2 - 0.0	-0.3±0.4	0.0 (-0.5 - 0.0)	-1.5 - 0.50	-0.6±0.6	-0.6 (-1.1 - 0.0)	-1.7 - 0.0	-0.4±0.6	0.0 (-0.9 - 0.0)
SR 12M	-1.7 - 0.75	-0.4±0.5	-0.2 (-1.0 - 0.0)	-1.5 - 0.75	-0.3±0.5	-0.1 (-0.5 - 0.0)	-1.5 - 0.50	-0.6±0.6	-0.5 (-1.2 - 0.0)	-1.7 - 0.0	-0.4±0.6	0.0 (-0.8 - 0.0)
CR baseline	23.2 - 28.1	25.2±1.0	25.0 (24.6 - 25.6)	23.2 - 28.1	24.9±1.0	24.8 (24.5 - 25.2)	23.3 - 26.7	25.2±0.8	25.2 (24.8 - 25.6)	23.7 - 28.1	25.5±1.2	25.1 (24.7 - 26.3)
CR 6M	23.6 - 28.1	25.2±0.9	25.1 (24.7 - 25.8)	23.6 - 28.1	24.9±0.9	24.9 (24.5 - 25.3)	23.8 - 27.2	25.4±0.8	25.2 (24.8 - 25.8)	23.7 - 28.0	25.4±1.2	25.2 (24.6 - 26.2)
CR 12M	23.5 - 28.2	25.3±1.0	25.2 (24.6 - 25.9)	23.5 - 28.2	25.0±0.9	25.1 (24.5 - 25.4)	24.0 - 27.4	25.5±0.8	25.3 (24.9 - 26.0)	23.7 - 28.2	25.5±1.3	25.2 (24.6 - 26.2)
AL baseline	-2.3 - 0.87	-0.3±0.5	-0.2 (-0.5 - 0.0)	-1.6 - 0.87	-0.2±0.4	-0.2 (-0.5 - 0.0)	-2.3 - 0.25	-0.5±0.5	-0.5 (-0.7 - -0.2)	-1.0 - 0.50	-0.1±0.3	-0.2 (-0.4 - 0.12)
AL 6M	-2.5 - 0.50	-0.3±0.4	-0.2 (-0.5 - 0.0)	-1.5 - 0.50	-0.2±0.4	-0.2 (-0.5 - 0.0)	-2.5 - 0.25	-0.5±0.6	-0.5 (-0.5 - 0.0)	-1.0 - 0.50	-0.1±0.3	-0.2 (-0.5 - 0.12)
AL 12M	-0.7 - 0.50	-0.0±0.2	0.0 (0.0 - 0.0)	-0.7 - 0.50	-0.0±0.3	0.0 (-0.2 - 0.0)	-0.5 - 0.25	-0.0±0.2	0.0 (0.0 - 0.0)	-0.5 - 0.25	-0.0±0.1	0.0 (0.0 - 0.0)
SER difference 12M	-0.1 - 0.67	0.16±0.1	0.14 (0.02 - 0.25)	-0.0 - 0.53	0.17±0.1	0.16 (0.08 - 0.21)	0.0 - 0.67	0.25±0.2	0.20 (0.10 - 0.43)	-0.1 - 0.31	0.04±0.1	0.02 (-0.0 - 0.10)
SR difference 12M	-1.0 - 0.50	-0.1±0.2	0.0 (-0.2 - 0.0)	-0.5 - 0.50	-0.0±0.2	0.0 (-0.2 - 0.0)	-1.0 - 0.12	-0.2±0.2	-0.2 (-0.5 - 0.0)	-0.5 - 0.37	-0.1±0.2	0.0 (-0.2 - 0.0)
CR difference 12M	-1.8 - 0.87	-0.2±0.3	-0.2 (-0.3 - 0.0)	-1.1 - 0.87	-0.2±0.4	-0.2 (-0.3 - 0.0)	-1.8 - 0.62	-0.2±0.4	-0.2 (-0.5 - 0.0)	-0.5 - 0.25	-0.0±0.2	0.0 (-0.2 - 0.0)
AL difference 12M	-1.0 - 0.50	-0.1±0.2	0.0 (-0.2 - 0.0)	-0.5 - 0.25	-0.0±0.1	0.0 (-0.2 - 0.0)	-1.0 - 0.25	-0.2±0.2	0.0 (-0.5 - 0.0)	-0.5 - 0.50	-0.0±0.2	0.0 (-0.2 - 0.0)

Table S1. Summary of refractive (SER, SR, CR) and biometric (AL) parameters of the left eye (LE) of each participant at baseline, 6 months, and 12 months for the total sample and by treatment group.

LE	SVL (n=20)	HAPD (n=21)	HAL (n=16)	Total	Chi square
AL decrease	1 (5%)	1 (4.8%)	4 (25%)	6 (10.5%)	P = 0.0537
AL increase	5 (25%)	10 (47.6%)	2 (12.5%)	17 (29.8%)	
Al stable	14 (70%)	10 (47.6%)	10 (62.5%)	34 (59.6%)	
Myopia improve	7 (35%)	4 (19%)	6 (37.5%)	17 (29.8%)	P = 0.4195
Myopia increase	4 (20%)	6 (28.6%)	1 (6.3%)	11 (19.3%)	
Myopia stable	9 (45%)	11 (52.4%)	9 (56.3%)	29 (50.9%)	

Table S2. Categorical distribution of axial length (AL) and spherical equivalent refraction (SER) changes in left eyes (LE) at 12 months by treatment group.

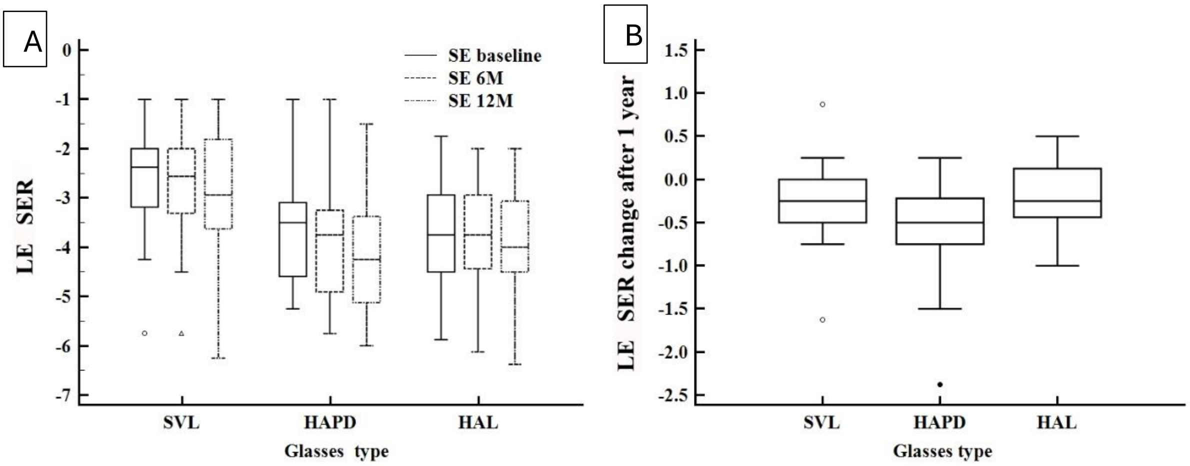

Figure S1. Changes in spherical equivalent refraction (SER) of the left eye (LE) over time and between treatment groups. A) Distribution of SER values at baseline, 6 months, and 12 months across the SVL, HAPD, and HAL groups. B) Total SER change after 12 months in each group.

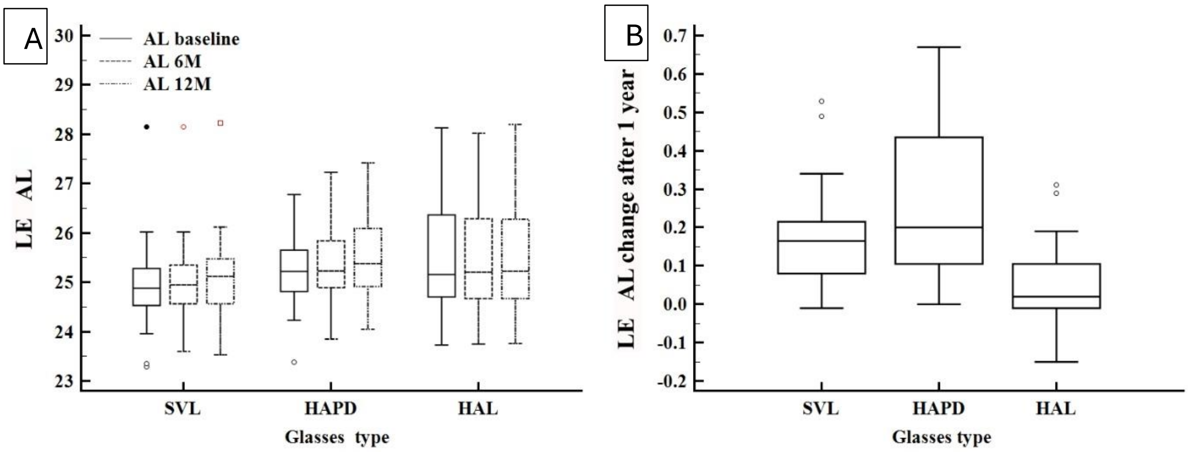

Figure S2. Changes in axial length (AL) of the left eye (LE) over time and between treatment groups. A) Distribution of AL values at baseline, 6 months, and 12 months across the SVL, HAPD, and HAL groups. B) Total AL change after 12 months by treatment group.
